# Regulation of *Pseudomonas* sp. PSC001 on the Artificial Rumen Environment Contaminated by Zearalenone

**DOI:** 10.3390/toxins17090471

**Published:** 2025-09-21

**Authors:** Yiming Han, Xinfeng Li, Xiaoli Ren, Chao Song, Zhaojie Zhang, Yufeng Gao, Dongmei Shi, Hongyu Deng, Heping Huangfu, Jinming Wang

**Affiliations:** 1 College of Veterinary Medicine, Henan University of Animal Husbandry and Economy, No. 6, Longzi Lake North Road, Zhengzhou 450046, China; hanyiming20002025@163.com (Y.H.); xinfengli@hnuahe.edu.cn (X.L.); renxiaoli@hnuahe.edu.cn (X.R.); hnuahesongchao@163.com (C.S.); zhaojiezhang@163.com (Z.Z.); yufenggao@163.com (Y.G.); dongmeishi126@126.com (D.S.); 80298@hnuahe.edu.cn (H.D.); 2 College of Veterinary Medicine, Shanxi Agricultural University, No. 1, Mingxian South Road, Taigu 030031, China; 3 Henan Key Laboratory of Unconventional Feed Resources Innovative, No. 6, Longzi Lake North Road, Zhengzhou 450046, China; 4 Zhengzhou Key Laboratory of Animal Nutrition Metabolic Diseases and Poisoning Diseases, No. 6, Longzi Lake North Road, Zhengzhou 450046, China

**Keywords:** zearalenone, *Pseudomonas*, artificial rumen simulation system, rumen environment

## Abstract

In this study, the RUSITEC system was used to study the regulation of rumen-derived *Pseudomonas* sp. PSC001 (PSC001) on the rumen environment contaminated by Zearalenone (ZEN). The rumen fluid of dairy cows was selected as the fermentation broth, and four experimental groups were set up: control group (CON), *Pseudomonas* group (PS), ZEN pollution group (ZEN), and PS and ZEN co-treatment group (PS + ZEN). The NH_3_-N, microbial protein (MCP), and volatile fatty acid (VFA) in the rumen fermentation broth were measured after culturing, and the changes in microbial community structure in rumen fluid were analyzed by 16S rRNA gene sequencing. After adding PSC001, the concentration of propionic acid, valeric acid, and butyric acid increased, and the acetate to propionate ratio and concentration of isovaleric acid decreased. ZEN exposure can lead to an abnormal increase in NH_3_-N, valeric acid, and isovaleric acid content and a decrease in MCP content. The content of NH_3_-N, valeric acid, and isovaleric acid decreased and the content of MCP increased in the PS + ZEN combined treatment group. The addition of PSC001 and ZEN significantly or extremely significantly increased the abundance of 18 genera and significantly or extremely significantly decreased the relative abundance of 5 genera in rumen fluid, respectively. It is worth noting that with the addition of both at the same time, the abundance of four genera in the PS + ZEN group was significantly or extremely significantly increased among the five genera with decreased abundance in the ZEN group. Among the 18 genera with increased abundance in the ZEN group, 10 genera in the PS + ZEN group decreased significantly or extremely significantly. In summary, the addition of PSC001 alleviated the negative impact of ZEN on the internal environment of rumen fermentation, and it also had a positive regulatory effect on rumen fermentation.

## 1. Introduction

Zearalenone (ZEN) is an estrogenic mycotoxin produced by Fusarium, which is widely found in moldy corn, wheat, and other grain feeds [1]. In recent years, with the influence of global warming and poor storage conditions, the problem of ZEN pollution in feed has become increasingly serious. Chhaya RS et al. [2] conducted a statistical analysis of 97 studies on mycotoxins such as ZEN in feed collected from the databases Web of Knowledge, Scopus, and Embase from 2011 to 2022. It was found that the detection rate of ZEN was 70%, and the concentration was 42.47–66.19 μg kg^−1^; Ching-Kuo Yang et al. [3] investigated the prevalence of mycotoxins in feed and feed ingredients in Taiwan Province of China from 2015 to 2017. The detection results of 820 corn flour and corn-based pig feed samples showed that the positive rate of ZEN reached 70.2%, second only to the first vomiting toxin (91.4%). In view of the abundant microbial resources in the rumen, ruminants are generally considered to have relatively strong mycotoxin tolerance, but some scholars have found that ZEN has a negative effect on ruminants. For example, after long-term intake of ZEN-contaminated diets in lactating cows, rumen osmotic pressure increased, 16 of 18 health indicators such as body temperature, respiratory rate, and heart rate showed negative regulation, and liver enzyme glutamate dehydrogenase decreased [4]. In vitro fermentation studies using an in vitro single-flow continuous culture system found that 10 mg/L ZEN concentration led to a decrease in NDF and ADF digestibility [5]. In addition, it is noteworthy that rumen microorganisms convert ZEN into more toxic metabolites [6,7], which will further aggravate the harm of ZEN to ruminants.

At present, the treatment methods for ZEN-contaminated feed can be divided into three categories: physical adsorption, chemical detoxification, and biodegradation [8,9,10]. Among them, the physical adsorption method is widely used because of its simple operation, but the commonly used adsorbents such as bentonite and yeast cell walls have clear limitations in practical application, mainly manifested as low adsorption efficiency and poor specificity [11]. Although chemical detoxification methods such as ozone treatment and ammoniation treatment can achieve better degradation effects, such methods often require special equipment support, and secondary pollutants may be produced during the treatment process [12]. In contrast, the microbial degradation method shows significant advantages; it not only has high degradation efficiency but also shows good specificity and environmental friendliness, so it is recognized as the most promising ZEN detoxification technology [13,14].

*Pseudomonas* is a large family with rich genetic diversity. More than 300 species have been identified [15]. Some *Pseudomonas* are pathogenic [16,17,18], but most of them are saprophytic and non-pathogenic [15]. Because *Pseudomonas* spp. are bacteria with strong environmental adaptability and metabolic diversity, they have broad application prospects in microbial fuel production, pollutant degradation, environmental protection, and ecological balance [19,20]. For example, it has been found that *Pseudomonas* from meat can inhibit the growth of *Escherichia coli* O157:H7 and reduce the expression of virulence genes of the E4 strain, thus weakening the pathogenicity of *Escherichia coli* O157:H7 [20]. Rumen-derived PS *stutzeri* MP4687 has the ability to degrade lignocellulosic biomass [21]. In recent years, the degradation characteristics of mycotoxins by this bacterium have also attracted the attention of some researchers [22,23]. In our previous study, a strain of *Pseudomonas sp PSC001* (PSC001) with ZEN degradation activity was isolated from the rumen fluid of healthy dairy cows. In the gavage test, it was found that it had no significant effect on the organ appearance change, organ index, and blood routine index of rats, and no visible pathogenicity was observed. In view of the fact that the strain is derived from the rumens of healthy dairy cows and has ZEN degradation activity, we speculate that it has a regulatory effect on the imbalance of the rumen internal environment caused by ZEN. The aim of this study is to evaluate the impact of PSC001 and/or ZEN on the rumen environment using rumen simulation technology (RUSITEC), and it will also explore the ecological regulatory effects both alone and in combination on the rumen.

## 2. Results

### 2.1. Effects of PSC001 and ZEN on Artificial Rumen Fermentation Parameters

Table 1 shows the fermentation changes in pH, NH_3_-N, and MCP in the RUSITEC system. The results showed that there was no significant change in pH between groups. Compared with the CON group, the NH_3_-N content of the PS group was significantly decreased (*p* < 0.01), the NH_3_-N content of the ZEN group was significantly increased (*p* < 0.01), and the MCP content was significantly decreased (*p* < 0.01). Compared with the ZEN group, the content of NH_3_-N in the PS + ZEN group was significantly decreased (*p* < 0.01), and the content of MCP was significantly increased (*p* < 0.01).

### 2.2. Effects of PSC001 and ZEN on VFA in Artificial Rumen

Table 2 shows the changes in volatile fatty acid (VFA) in the artificial rumen. The results showed that compared with the CON group, the concentrations of propionic acid, valeric acid, and butyric acid in the PS group were significantly or extremely significantly increased (*p* < 0.05, *p* < 0.01), and the acetate to propionate ratio and concentration of isovaleric acid were extremely significantly decreased (*p* < 0.01). The concentrations of valeric acid, isovaleric acid, butyric acid, and isobutyric acid in the ZEN group were significantly or extremely significantly increased (*p* < 0.05, *p* < 0.01). Compared with the ZEN group, the concentrations of valeric acid and isovaleric acid in the PS + ZEN group were significantly or extremely significantly decreased (*p* < 0.05, *p* < 0.01).

### 2.3. Effects of PSC001 and ZEN on the Microbes in Artificial Rumen

Table 3 shows the changes in rumen microflora at the level of 16S rRNA gene phylum classification. A total of six phyla were identified, of which Firmicutes, Bacteroidota, and Proteobacteria were the main dominant phyla with an abundance of more than 10%. The results showed that compared with the CON group, the relative abundance of Firmicutes and Actinobacteria in the PS group and the ZEN group increased significantly (*p* < 0.01), the relative abundance of Proteobacteria decreased significantly (*p* < 0.01), and the relative abundance of Spirochaetes in the ZEN group increased significantly (*p* < 0.01). Compared with the ZEN group, the relative abundance of Proteobacteria in the PS + ZEN group was significantly increased (*p* < 0.01), and the relative abundance of Spirochaetes and Actinobacteria was significantly or extremely significantly decreased (*p* < 0.05, *p* < 0.01).

Table 4 shows the changes in rumen fluid microflora at the level of 16S rRNA gene genus classification, where a total of 34 were identified. The results showed that compared with the CON group, the relative abundance of 18 genera in the PS group was significantly or extremely significantly increased (*p* < 0.05, *p* < 0.01), and the relative abundance of 5 genera was significantly or extremely significantly decreased (*p* < 0.05, *p* < 0.01). The relative abundance of 18 genera in the ZEN group was significantly or extremely significantly increased (*p* < 0.05, *p* < 0.01), and the relative abundance of 5 genera was significantly or extremely significantly decreased (*p* < 0.05, *p* < 0.01). Compared with the ZEN group, among the five genera with decreased abundance in the ZEN group, the abundance of four genera in the PS + ZEN group increased significantly or extremely significantly (*p* < 0.05, *p* < 0.01), and the abundance of one genus decreased extremely significantly (*p* < 0.01). Among the 18 genera with increased abundance in the ZEN group, 10 genera in the PS + ZEN group were significantly or extremely significantly decreased (*p* < 0.05, *p* < 0.01), and 6 genera were significantly or extremely significantly increased (*p* < 0.05, *p* < 0.01).

## 3. Discussion

pH is an important index to observe rumen acidosis and homeostasis, which can reflect the environmental characteristics of the rumen and the fermentation mode of feed in the rumen. Hartinger et al. [24] found that ZEN pollution may lead to a decrease in rumen pH in dairy cows by affecting microflora and VFA. The results of this study were similar to those of Hartinger’s study. The pH decreased after ZEN exposure, but the difference was not significant and was still within the normal range. This may be related to the neutralization brought by the continuous introduction of buffer into the fermentation tank. From another point of view, the relative stability of the pH value indicates that the artificial rumen simulation system used in the experiment can provide a relatively stable fermentation condition, which is helpful for evaluating other rumen fermentation parameters more objectively.

NH_3_-N is the main product of digestion and metabolism of protein organic matter in the rumen, and its concentration can reflect the degree of protein degradation in rumen fermentation substrate [25]. Wang et al. [26] showed that the content of NH_3_-N in the rumens of dairy cows increased after feeding diets contaminated with ZEN and AFB1. Consistently, in this study, ZEN exposure increased the content of NH_3_-N, and the NH_3_-N level decreased significantly when PSC001 and ZEN were added at the same time. Rumen microorganisms can not only use NH_3_-N to synthesize MCP but also generate NH_3_-N by decomposing protein in feed. Efficient ruminant productivity requires the best protein in the feed, and animal productivity can be improved by simultaneously increasing the rumen availability of carbohydrates and proteins [27]. In this study, ZEN exposure significantly reduced the MCP content; when PSC001 and ZEN were added at the same time, the content of MCP in the fermentation broth increased. The above results indicate that the addition of PSC001 alleviates the negative effects of ZEN by reducing the NH_3_-N content and regulating MCP levels, thereby helping to improve nutrient digestibility and rumen fermentation.

VFA is a class of short-chain fatty acid with a carbon chain length between two and six carbon atoms. It is produced by rumen microorganisms fermenting cellulose, hemicellulose, and other carbohydrates, including acetic acid, propionic acid, and butyric acid, etc. It is an important part of ruminant energy needs [28,29]. The higher proportion of acetic acid usually reflects the strong activity of cellulolytic bacteria, which is suitable for roughage digestion, but the energy utilization rate is low. The higher proportion of propionic acid indicates that the utilization of easy-to-ferment carbohydrate is enhanced. Propionic acid, as the main sugar precursor, can provide energy for the host more efficiently and improve the feed conversion rate [30,31]. The results of this study showed that the concentration of propionic acid in rumen fluid of the PS group increased and the ratio of acetic acid to propionic acid decreased, suggesting that PSC001 promoted the transformation of the rumen fermentation mode to the propionic acid fermentation mode and improved energy utilization efficiency.

The decrease in isovaleric acid content may be related to the *liuRABCDE* gene cluster system of *Pseudomonas*; this contains a complete metabolic enzyme system involved in the catabolism of leucine, which may reduce the supply of precursors for isovaleric acid synthesis [32]. The contents of valeric acid, isobutyric acid, and isovaleric acid in the ZEN group were significantly increased, which was similar to the results of Hartinger et al. [28]. This may be because ZEN affects the metabolism of branched-chain amino acids, thereby increasing the contents of branched-chain fatty acids. Isobutyric acid and isovaleric acid are short-chain volatile fatty acids (Isoacid) containing 4–5 carbon atoms. Studies have shown that isovaleric acid reduces the effective degradation rate of soybean meal crude protein in the rumens of cattle [33]. In this study, the concentrations of valeric acid and isovaleric acid in the PS + ZEN group were lower than those in the ZEN group, indicating that the addition of PSC001 could inhibit the excessive increase in branched-chain fatty acids caused by ZEN. In summary, PSC001 can increase the content of propionic acid, reduce the ratio of acetic acid to propionic acid, and regulate the increase in partial isoacid concentration caused by ZEN, which is conducive to optimizing rumen energy distribution and improving the utilization rate of feed by the host. It provides a new theoretical basis for regulating the nutritional metabolism of ruminants.

At the level of phylum classification, Firmicutes, Bacteroidetes, and Proteobacteria were the dominant flora in the control group. After adding PSC001 and ZEN, the abundance of Firmicutes and Proteobacteria changed significantly, but their dominant flora status did not change. Firmicutes is the dominant phylum in the rumen, which has the effect of degrading crude fiber [34,35]; the continuous increase in the abundance of Proteobacteria is considered to be a sign of intestinal flora imbalance [36]. The abundance of Firmicutes in the PS group was significantly higher than that in the control group, and the abundance of Proteobacteria was significantly lower than that in the control group, indicating that PSC001 may help promote the degradation of crude fiber and may have the effect of promoting the recovery of rumen flora. The abundance of Firmicutes and Proteobacteria in the ZEN group showed the same trend as that of the PS group, indicating that the effect of ZEN on the rumen is more complex. Whether it also has the effect of promoting the decomposition of crude fiber and promoting the recovery of rumen flora needs further study. The abundance of Proteobacteria in the PS + ZEN group was significantly higher than that in the PS group and the ZEN group, the abundance of Spirochaetes was significantly lower than that in the ZEN group, the abundance of Actinobacteria was significantly lower than that in the PS group and the ZEN group, and the abundance of Firmicutes was also lower than that in the PS group and the ZEN group, indicating that the simultaneous addition of PSC001 and ZEN had an antagonistic effect on the abundance of bacteria. It was speculated that it could be caused by the degradation of ZEN by PSC001.

At the genus level, compared with the control group, the abundance of 18 genera in the PS group and the ZEN group increased significantly or extremely significantly, and the relative abundance of 5 genera decreased significantly or extremely significantly, indicating that PSC001 and ZEN had an effect on the microbial community structure of rumen fluid. After adding PSC001 and ZEN at the same time, among the five genera with decreased abundance in the ZEN group, the abundance of four genera in the PS + ZEN group increased significantly or extremely significantly. Among the 18 genera with increased abundance in the ZEN group, 10 genera in the PS + ZEN group decreased significantly or extremely significantly. The results reflected that the addition of PSC001 had a certain negative regulatory effect on the change in rumen bacteria population structure caused by ZEN. At the same time, it was found that *Prevotella* was the dominant genus in each group, which was consistent with the findings of other researchers [37,38]. In this study, it was observed that the relative abundance of *Prevotella* decreased significantly after ZEN exposure. *Prevotella* is an important fiber-degrading bacterium in the rumen [39], and its decreased abundance may affect the fiber degradation function of the rumen. It is worth noting that the abundance of *Prevotella* in the PS + ZEN group was significantly increased, indicating that the addition of PSC001 could improve the inhibitory effect of ZEN on Prevotella. *Succinivibrionaceae_UCG-002* is also one of the dominant genera in the control group. Cui, Y. [40] showed that *Succinivibrionaceae_UCG-002* was negatively correlated with glycan biosynthesis metabolism, cofactor and vitamin metabolism, and nucleotide metabolism and translation. The abundance of the genus decreased significantly after the addition of PSC001 or ZEN, indicating that the two may have an inhibitory effect on carbohydrate, cofactor, and vitamin metabolism and nucleotide metabolism, suggesting the complexity of PSC001 in rumen regulation; when PSC001 and ZEN were added at the same time, the abundance of *Succinivibrionaceae_UCG-002* in rumen fluid increased significantly, which may be due to the degradation of ZEN by PSC001, which antagonized the effects of PSC001 and ZEN. *Kurthia* belongs to Planococcaceae, Firmicutes, which is widely distributed in nature. It can be isolated from biogas slurry, sewage, the oral cavities of deer, and the rumen fluid of Holstein cattle [41,42,43,44]. In recent years, the reports of *Kurthia* have gradually increased, mostly focusing on the prevention and control of pollutants, antibiotic degradation, stress resistance, etc. [45]. Of course, some studies have shown that the bacteria have conditional pathogenicity, such as Lozica, L, etc. [46]. It was found that *Kurthia* does not cause primary infection but may have opportunistic pathogenicity to birds. *Kurthia* was also one of the dominant genera in the control group. Compared with the control group, the abundance of *Kurthia* in the PS group and the PS + ZEN group decreased significantly, and the abundance of *Kurthia* in the ZEN group did not change significantly, indicating that PSC001 had a significant inhibitory effect on Kurthia, and it was speculated that it had the potential to inhibit its pathogenicity.

## 4. Conclusions

PSC001 significantly ameliorated ZEN-induced protein metabolic disorders by reducing NH_3_-N concentrations and enhancing MCP synthesis efficiency. Furthermore, PSC001 optimized energy utilization efficiency by promoting propionic acid production, lowering the acetate to propionate ratio, and simultaneously suppressing the abnormal increase in branched-chain fatty acids, such as valeric and isovaleric acids, induced by ZEN. Moreover, PSC001 modulated the rumen microbial community structure through ZEN antagonism, restoring the abundance of fiber-degrading bacteria Prevotella and succinate-producing Vibrio_UCG-002, thereby reversing the adverse effects of ZEN on rumen microbiota. In summary, PSC001 can effectively antagonize the toxicity of ZEN by regulating the rumen microbial structure and metabolic function through multiple targets, which provides a theoretical basis for the development of mycotoxin detoxification strategies in ruminants based on microbial regulation.

## 5. Materials and Methods

### 5.1. Experimental Animals and Experimental Design

The donors of rumen fluid were four Simmental beef cattle from a slaughterhouse in Xingyang County. The weight of the experimental animals was about 450 kg, and the age was about 18 months. After rumen fluid collection, it was deployed to the RUSITEC system for in vitro fermentation determination. The diet and feeding amount of rumen fluid donors met the nutritional standards specified in the ‘Chinese Beef Cattle Feeding Standards’ (NY/T815-2004). The rumen fluid was collected from commercial slaughterhouses and did not involve live animal experiments, in line with China’s animal ethics regulations. The dietary composition of cattle is shown in Table 5. PSC001 was screened from bovine rumen fluid by the Zhengzhou Key Laboratory of Animal Nutrition and Metabolic Diseases and Poisoning Diseases in the early stage. Studies have shown that it has significant ability to degrade ZEN. At present, the strain has been submitted to the Chinese Typical Microorganism Preservation Center, and the conservation number is CCTCC NO:M2023814. ZEN toxin was purchased from WITEGA (product number: MT001-10 mg, CAS 17924-92-4). In this experiment, a two-factor experimental design was used, with PSC001 and ZEN as the two experimental factors. The experiment was divided into three cycles, and each cycle was continuously cultured for 7 days. Four treatment groups were set up: the experiment:control group (CON), *Pseudomonas* group (PS), ZEN group (ZEN), and *Pseudomonas* and ZEN combined treatment group (PS + ZEN). The adaptation period was 3 days before the experiment, and 20 g of feed without any other components was fed at 8:00 a.m. and 8:00 p.m. every day. After the end of the adaptation period, feeding adjustment was carried out according to the treatment group: at 8:00 in the morning, for the ZEN group, 1 mL of 5 mg/mL ZEN was evenly sprayed on the feed, for the PS group, we added 5 mL of 4 × 10^8^ CFU/mL PSC001 solution per day on the basis of TMR. After 30 min of volatilization in the dark, it was added to the rumen simulator, and 5 mL of sterile buffer was added. The PS + ZEN group had the same amount of *Pseudomonas* solution and the same amount of ZEN, and the CON group added the same amount of sterile buffer. At 8:00 p.m., each group was only fed with conventional TMR feed. Samples were taken from the fermentation tank on the 7th day of each test period, and the samples were stored at −80 °C after solid–liquid separation.

### 5.2. Simulated Rumen Fermentation Technology

The rumen simulation technique (RUSITEC, AR III-04-06, Changsha Zisen Biotechnology Co., Ltd., Changsha, China) consists of a fermenter, a thermostatic water jacket, a stirring device, a buffer input system, a gas collection device, and an overflow system. The fermentation tank simulated the rumen environment, and the constant-temperature water maintained the body temperature condition of 39 °C. The stirring device simulated the rumen contraction movement through different rotation speeds (5–25 rpm), and the buffer input system continued to provide nutrition and maintain pH stability. The system can accurately control the fermentation conditions and is widely used to study the effects of rumen movement on microbial fermentation, gas production, and nutrient digestion. Before the formal experiment, the sealing of the RUSITEC system must be ensured, and the trial operation must be carried out. During the formal experiment, the flask was first preheated to 39 °C and filled with CO_2_. After the cattle were slaughtered, the rumen fluid was quickly collected into a thermos flask, filtered through four layers of disinfected gauze, and sent back to the laboratory. The anaerobic environment was maintained by long-term introduction of N_2_ gas, and 500 mL of preheated filtered rumen fluid and 500 mL of McDougall [47] buffer were introduced into each fermenter. After the equipment is running, inject N2 gas into each fermentation tank while maintaining a temperature of 39 ± 0.5 °C, and then add 20 g of fermentation substrate (DM based). The composition of the fermentation substrate was consistent with the feed composition of the rumen fluid donor cows. After the feed was brought back from the dairy farm, it was dried to a constant weight in a constant-temperature drying oven at 65 °C, and then it was ground using a grinding machine through a 1 mm aperture sieve. Through the intelligent control system, the system uses the stepper motor to stir the contents of the fermentation tank at a speed of 25 r/min. The McDougall buffer is fed into the fermenter at a rate of 6% per hour through a pressure pump. At the same time, the catheter of the overflow bottle is monitored to ensure that the overflow liquid and the undegraded solid-phase fermentation substrate in each fermentation tank can be collected in time and terminated in the overflow bottle.

### 5.3. The Counting and Treatment of Pseudomonas

PSC001 was inoculated into 100 mL broth medium and cultured at 37 °C 160 r/min for 24 h. The viable count was 1 × 10^9^ CFU/mL. The culture solution (80 mL) was centrifuged at 5000 rpm for 5 min. After centrifugation, the precipitate was mixed with 5 mL sterile PBS buffer and added to the fermenter.

### 5.4. The Configuration of ZEN Working Fluid

We dissolved 10 mg of ZEN in 10 mL of methanol to prepare a mother liquid of 1 mg/mL, and 1 mL was diluted 10 times with methanol to prepare a working liquid of 0.1 mg/mL.

### 5.5. Determination of Environmental Indicators in Artificial Rumen

#### 5.5.1. Determination of pH

After collecting the rumen fluid on the last day of fermentation, the pH was measured using a portable pH meter (Testo206-pH, Shenzhen Testo Instrument Co., Ltd., Shenzhen, China).

#### 5.5.2. Determination of NH_3_-N, MCP, and VFA

A multifunctional microplate reader (Synergy HT, Burten Instrument Co., Ltd., Vemont, VT, USA) was used to detect ammonia nitrogen (NH_3_-N) and microbial protein (MCP) in rumen fluid by a phenol-oxygen assay and the Coomassie brilliant blue method [48,49], referring to the test method of Olagunju L K [50], and the volatile fatty acids were determined by gas chromatography (GC-9790 plus, Zhejiang Welfare Analytical Instrument Co., Ltd., Taizhou, China).

#### 5.5.3. Detection of Microbial Community Composition in Rumen Fluid

DNA was extracted from the rumen fermentation broth on the final day of culture using a Fecal DNA Extraction Kit (B618763-0100, Shanghai Biotech Co., Ltd., Shanghai, China). Qualified DNA (with a D_260_/D_280_ ratio between 1.7 and 1.9) was sent to Shanghai Biotech Co., Ltd., for sequencing. The V3-V4 region of the bacterial 16S rRNA gene was amplified via PCR using primers 341F (5′-CCTACGGNGGCWGCAG-3′) and 805R (5′-GACTACHVGGGTATCTAATCC-3′). The first round of PCR amplification was performed using universal primers in a 30 μL reaction system on a PCR instrument (ETC 811, Beijing Dongsheng Innovation Biotechnology Co., Ltd., Beijing, China). The reaction mixture contained 15 μL of 2 × Hieff^®^ Robust PCR Master Mix (Yeasen, 10105ES03), 1 μL of each forward and reverse primer, 10–20 ng of template DNA, and 9–12 μL of water. The cycling parameters were as follows: First, there was a 3 min pre-denaturation step at 94 °C, followed by five cycles of 94 °C for 30 s, 45 °C for 20 s, and 65 °C for 30 s. Then, 20 cycles were performed at 94 °C for 20 s, 55 °C for 20 s, and 72 °C for 30 s, concluding with a 5 min extension at 72 °C. Then, we performed a second round of amplification using Illumina bridge PCR-compatible primers. The 30 μL reaction mixture contained 15 μL of 2× Hieff^®^ Robust PCR Master Mix (Yeasen, 10105ES03), 1 μL of each forward and reverse primer,10–20 ng of template DNA, and 9–12 μL of H_2_O. The cycling parameters were a 95 °C pre-denaturation for three minutes, followed by five cycles of 94 °C for 20 s, 55 °C for 20 s, and 72 °C for 30 s. The reaction was terminated with a 5 min extension at 72 °C. Library fragment sizes were analyzed via 2.0% agarose gel electrophoresis. The library’s quality was assessed using the Qubit 3.0 Fluorometer System (Q33216, Thermo Fisher Scientific Co., Ltd. Shanghai, China).

### 5.6. Statistical Analysis of Data

Statistical analysis was performed using SPSS 27.0 statistical software, with PSC001 and ZEN as the two experimental factors. One-way analysis of variance (one-way ANOVA) and Duncan’s multiple comparisons were used to test the data. The results were expressed as the standard error (SEM) and the significance level (*p*), with *p* < 0.05 indicating a significant difference and *p* < 0.01 indicating an extremely significant difference.

## Figures and Tables

**Table 1:** Artificial rumen fermentation parameters.

Item	Group					
	CON	PS	ZEN	PS + ZEN	SEM	*p*
pH	6.45	6.37	6.37	6.72	0.01	0.62
NH_3_-N/(mg/L)	126.08 ^A^	119.24 ^B^	150.29 ^C^	116.59 ^B^	0.40	<0.001
MCP/(mg/L)	9.37 ^Ab^	9.52 ^Aab^	8.83 ^Bc^	9.56 ^Aa^	0.01	<0.001

In the same row, different superscripts on lowercase letters indicate significant differences (*p* < 0.05), different superscripts on uppercase letters indicate extremely significant differences (*p* < 0.01), and the same or different superscripts on uppercase letters indicate insignificant differences (*p* < 0.05).

**Table 2:** VFA content in artificial rumen mg/mL.

Item	Group					
	CON	PS	ZEN	PS + ZEN	SEM	*p*
Acetic	1.84	2.01	2.10	2.17	0.09	0.69
Propionic	0.98 ^a^	1.38 ^b^	1.04 ^a^	1.13 ^ab^	0.06	0.03
Valeric	0.13 ^Aa^	0.38 ^Bb^	0.25 ^Ac^	0.20 ^Bd^	0.03	<0.001
Butyrate	0.70 ^A^	0.90 ^B^	0.96 ^B^	0.95 ^B^	0.04	0.004
Isobutyric	0.047 ^A^	0.047 ^A^	0.054 ^B^	0.052 ^B^	0.001	0.003
Isovaleric	0.16 ^A^	0.13 ^B^	0.18 ^C^	0.16 ^A^	0.01	<0.001
Acetic/Propionic	1.88 ^A^	1.45 ^B^	2.01 ^A^	1.91 ^A^	0.07	<0.001
TVFA	3.86	4.84	4.58	4.66	0.18	0.22

In the same row, different superscripts on lowercase letters indicate significant differences (*p* < 0.05), different superscripts on uppercase letters indicate extremely significant differences (*p* < 0.01), and the same or different superscripts on uppercase letters indicate insignificant differences (*p* < 0.05).

**Table 3:** 16S-alpha gate classification level.

Item	Group					
	CON	PS	ZEN	PS + ZEN	SEM	*p*
Firmicutes	39.91 ^Aa^	48.98 ^Bb^	47.19 ^Bbc^	43.72 ^Bac^	1.17	0.003
Bacteroidota	31.85	34.58	34.07	36.60	0.70	0.10
Proteobacteria	23.77 ^A^	8.11 ^B^	11.39 ^C^	14.65 ^D^	1.77	<0.001
Spirochaetota	0.82 ^Aa^	1.03 ^Aa^	2.07 ^Bb^	1.41 ^Ba^	0.16	0.01
Actinobacteriota	0.23 ^A^	3.21 ^B^	2.41 ^C^	1.32 ^D^	0.34	<0.001
Verrucomicrobiota	1.16	0.95	0.65	0.48	0.12	0.17
Other	2.26 ^Aa^	3.15 ^Bb^	2.22 ^Aa^	1.81 ^Ac^	0.15	<0.001

In the same row, different superscripts on lowercase letters indicate significant differences (*p* < 0.05), different superscripts on uppercase letters indicate extremely significant differences (*p* < 0.01), and the same or different superscripts on uppercase letters indicate insignificant differences (*p* < 0.05).

**Table 4:** 16S-alpha genus classification level.

Item	Group					
	CON	PS	ZEN	PS + ZEN	SEM	*p*
*Prevotella*	23.31 ^ab^	19.99 ^bc^	19.00 ^c^	24.25 ^a^	0.82	0.03
*Succinivibrionaceae_UCG-002*	16.16 ^A^	1.60 ^B^	3.74 ^C^	5.33 ^D^	1.7	<0.001
*Succinivibrio*	5.02 ^Aa^	5.06 ^Ab^	5.96 ^Ac^	7.49 ^Bc^	0.32	<0.001
*Rikenellaceae_RC9_gut_group*	3.66 ^Aa^	5.83 ^Bb^	4.85 ^Ac^	4.00 ^Ad^	0.26	<0.001
*Kurthia*	11.63 ^Aa^	2.51 ^Bb^	10.60 ^Aa^	2.01 ^Bb^	1.46	0.002
*Treponema*	0.72 ^Aa^	0.89 ^Aa^	1.94 ^Bb^	1.26 ^Ba^	0.16	0.004
*Succiniclasticum*	4.43 ^Aa^	2.74 ^Bc^	3.56 ^Ab^	3.10 ^Bbc^	0.21	<0.001
*Christensenellaceae_R-7_group*	1.97 ^Aa^	3.77 ^Bb^	3.52 ^Bb^	2.78 ^Ac^	0.22	<0.001
*NK4A214_group*	1.73 ^Aa^	4.31 ^Bb^	3.07 ^Ac^	1.97 ^Aa^	0.32	<0.001
*Ruminococcus*	1.18 ^Aa^	2.80 ^Bb^	1.23 ^Aa^	1.34 ^Aa^	0.21	<0.001
*Clostridium_sensu_stricto_1*	0.61 ^A^	4.44 ^B^	1.16 ^A^	0.93 ^A^	0.47	<0.001
*norank_F082*	1.11 ^Aa^	2.73 ^Bb^	2.21 ^Ac^	1.27 ^Aa^	0.2	<0.001
*norank_Muribaculaceae*	0.79 ^Aa^	1.36 ^Ab^	2.26 ^Bc^	1.79 ^Ad^	0.17	<0.001
*norank_Clostridia_UCG-014*	1.18 ^Aa^	2.12 ^Bb^	1.54 ^Ac^	1.31 ^Aac^	0.12	<0.001
*Butyrivibrio*	1.01	1.26	0.68	1.21	0.13	0.414
*UCG-005*	1.12 ^Aa^	2.58 ^Bb^	1.72 ^Ac^	1.34 ^Aa^	0.17	<0.001
*Lachnospiraceae_XPB1014_group*	1.18 ^Aa^	2.12 ^Bb^	0.89 ^Ac^	1.52 ^Ad^	0.14	<0.001
*possible_genus_Sk018*	0.78 ^Aa^	1.49 ^Bbc^	1.25 ^Ab^	1.63 ^Bc^	0.1	<0.001
*Eubacterium_ruminantium_group*	0.65 ^Aa^	0.46 ^Ab^	0.81 ^Ac^	2.44 ^Bd^	0.24	<0.001
*Saccharofermentans*	1.20 ^Aa^	1.41 ^Ab^	1.78 ^Bc^	0.70 ^Ad^	0.12	<0.001
*Prevotellaceae_UCG-001*	0.74 ^Aa^	1.20 ^Bb^	0.82 ^Aa^	0.84 ^Aa^	0.05	<0.001
*Bifidobacterium*	0.05 ^A^	2.32 ^B^	1.33 ^C^	1.11 ^C^	0.25	<0.001
*Shuttleworthia*	0.08 ^Aa^	0.12 ^Aa^	0.49 ^Bb^	1.17 ^Ac^	0.13	<0.001
*UCG-002*	0.51 ^Aa^	0.59 ^Aa^	0.61 ^Ba^	0.86 ^Bb^	0.05	0.01
*norank_WCHB1-41*	0.91	0.71	0.52	0.35	0.09	0.15
*Prevotella_7*	0.08 ^Aa^	0.11 ^Aa^	1.82 ^Ab^	2.62 ^Bc^	0.33	<0.001
*Pseudobutyrivibrio*	0.62 ^A^	0.24 ^B^	0.24 ^B^	0.59 ^A^	0.06	<0.001
*norank_Eubacterium_coprostanoligenes_group*	0.41 ^Aa^	0.98 ^Bb^	0.68 ^Ac^	0.51 ^Aa^	0.07	<0.001
*Lachnospira*	0.05 ^A^	0.13 ^A^	0.14 ^A^	1.24 ^B^	0.15	<0.001
*norank_Selenomonadaceae*	0.02 ^A^	0.17 ^A^	0.07 ^A^	1.76 ^B^	0.22	<0.001
*Olsenel*	0.07 ^A^	0.67 ^B^	0.86 ^B^	0.08 ^A^	0.11	<0.001
*Solibacillus*	0.08	0.01	0.01	0.26	0.05	0.20
*Lysinibacillus*	0.01	0.001	0.01	0.84	0.15	0.10
*unclassified_Lachnospiraceae*	2.29 ^Aa^	2.75 ^Ab^	2.54 ^Aab^	4.22 ^Bc^	0.23	<0.001
*Other*	14.63 ^Aa^	20.52 ^Ab^	18.06 ^Bc^	15.91 ^Aa^	0.7	<0.001

In the same row, different superscripts on lowercase letters indicate significant differences (*p* < 0.05), different superscripts on uppercase letters indicate extremely significant differences (*p* < 0.01), and the same or different superscripts on uppercase letters indicate insignificant differences (*p* < 0.05).

**Table 5:** Diet composition and nutritional level (dry matter basis).

Ingredients	Contents	Nutrient Components	Contents
Corn silage	49.60	NEL/(MJ/kg) ^②^	6.64
Pressed corn	14.60	DM	95.70
Bean meal	10.30	CP	13.30
Brewery mash	8.30	EE	3.66
Moisture	6.20	Ca	0.79
Domestic oats	3.30	P	0.38
Imported alfalfa	4.10	NDF	33.30
Wheat bran	1.80	ADF	22.60
Premix ^①^	1.40		
NaHCO_3_	0.40		
Total	100.00		

① Premix: VA 800,000 IU; VD_3_ 180,000 IU; VE 15,000 IU; copper 680 mg; zinc 1800 mg; manganese 1350 mg; iodine 40 mg; cobalt 20 mg; selenium 30 mg. ② Net energy of milk production was a calculated value, and other indexes were measured values.

## Data Availability

The original contributions presented in this study are included in the article. Further inquiries can be directed to the corresponding author(s).
